# Comprehensive analysis of single molecule sequencing-derived complete genome and whole transcriptome of *Hyposidra talaca* nuclear polyhedrosis virus

**DOI:** 10.1038/s41598-018-27084-y

**Published:** 2018-06-12

**Authors:** Thong T. Nguyen, Kushal Suryamohan, Boney Kuriakose, Vasantharajan Janakiraman, Mike Reichelt, Subhra Chaudhuri, Joseph Guillory, Neethu Divakaran, P. E. Rabins, Ridhi Goel, Bhabesh Deka, Suman Sarkar, Preety Ekka, Yu-Chih Tsai, Derek Vargas, Sam Santhosh, Sangeetha Mohan, Chen-Shan Chin, Jonas Korlach, George Thomas, Azariah Babu, Somasekar Seshagiri

**Affiliations:** 1 Department of Molecular Biology, Genentech Inc., 1 DNA WAY, South San Francisco, CA 94080 USA; 2 AgriGenome Labs Private Limited, 501, SCK01 Building, SmartCity Kochi, Infopark Road, Kakkanad, Kochi, Kerala 682 042 India; 3 SciGenom Research Foundation, 3rd Floor, Narayana Health City, #258/A, Bommasandra, Hosur Road, Bangalore, Karnataka 560 099 India; 4 Department of Pathology, Genentech Inc, 1 DNA WAY, South San Francisco, CA 94080 USA; 5 Tea Research Association, North Bengal Regional R & D Centre, Nagrakata, Jalpaiguri, West Bengal 735 225 India; 6 Pacific Biosciences, 1305O’Brien Dr, Menlo Park, CA 94025 USA; 7 SciGenom Labs Pvt Ltd, Plot no: 43A,SDF, 3rd floor, A Block, CSEZ, Kakkanad, Kochi, Kerala 682 037 India

## Abstract

We sequenced the *Hyposidra talaca* NPV (HytaNPV) double stranded circular DNA genome using PacBio single molecule sequencing technology. We found that the HytaNPV genome is 139,089 bp long with a GC content of 39.6%. It encodes 141 open reading frames (ORFs) including the 37 baculovirus core genes, 25 genes conserved among lepidopteran baculoviruses, 72 genes known in baculovirus, and 7 genes unique to the HytaNPV genome. It is a group II alphabaculovirus that codes for the F protein and lacks the *gp64* gene found in group I alphabaculovirus viruses. Using RNA-seq, we confirmed the expression of the ORFs identified in the HytaNPV genome. Phylogenetic analysis showed HytaNPV to be closest to BusuNPV, SujuNPV and EcobNPV that infect other tea pests, *Buzura suppressaria*, *Sucra jujuba*, and *Ectropis oblique*, respectively. We identified repeat elements and a conserved non-coding baculovirus element in the genome. Analysis of the putative promoter sequences identified motif consistent with the temporal expression of the genes observed in the RNA-seq data.

## Introduction

Tea is a widely consumed beverage. India is the second largest producer of Tea^1^. During 2015–16 India saw a record tea production of 1,233 million kg and it exported 230 million kg valued at ~700 million USD^1^. A majority of the tea is cultivated in north-eastern states of Assam (52.0%) and West Bengal (25.8%) in India. Recently, *Hyposidra talaca* (Walk.) (Lepidoptera: Geometridae), typically found in forests of north-east India, has become a major defoliating pest of tea in these regions, surpassing *Buzura suppressaria* (Guen.) (Lepidoptera: Geometridae)^2–4^. Current management of *H*. *talaca* involves the use of chemical pesticides that include organophosphates and synthetic pyrethroids^2,5^.

Nuclear polyhedrosis viruses (NPVs) and granulosis viruses (GVs) are baculoviruses that infect insects^6^. Over 600 different baculoviruses have been reported^6^. Baculovirus double stranded circular DNA genomes range from 80 to 180 kb in size and encode between 90 and 180 genes^6^. The baculoviridae family consists of viruses that infect Lepidopterans (*Alphabaculoviruses* (NPVs) and *Betabaculoviruses* (GVs)), Hymenopterans (*Gammabaculoviruses* (NPVs)) and Dipterans (*Deltabaculoviruses* (NPVs)). The *Alphabaculoviruses* are further classified into group I and group II based on phylogenetic analysis and the presences of either g64 fusion protein (found in group I) or the F protein (found in group II)^6^.

Recently, HytaNPV, a naturally occurring baculovirus, was reported to be effective against *H*. *talaca*^7–9^. We have applied PacBio single-molecule sequencing to obtain the complete sequence of HytaNPV. Also, we sequenced the RNA from infected larvae to obtain the whole viral transcriptome. Using an integrated analysis strategy, we have combined the genome and transcriptome data and performed a comparative analysis to annotate and understand the HytaNPV genome.

## Results

### HytaNPV isolation and characterization

**Figure 1:**
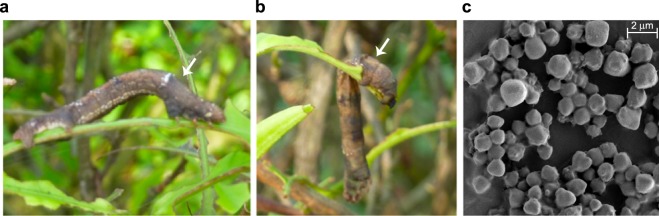
(**a**) *H*. *talaca* healthy larvae. (**b**) *H*. *talaca* larvae exhibiting typical NPV infected symptom hanging from tea leaves. (**c**) Scanning electron micrograph showing the polyhedral inclusion bodies.

We obtained *H*. *talaca* infected larvae that showed typical symptoms of NPV infection (Fig. 1a-b) from tea fields of Dooars region in West Bengal, India. The infected larval cadavers were processed to obtained purified virus particles. We examined the virus particles using an electron microscope and confirmed the presence of polyhedral inclusion bodies (Fig. 1c).

### Single molecule sequencing and analysis of HytaNPV Genome

We sequenced HytaNPV DNA using PacBio single molecule sequencing^10^. A total of 124,978 reads with an average length of 8,635 bp was obtained. *De novo* assembly of the reads yielded a circular genome of ~139 kb with the overall coverage of 2,237×. We also generated sequence data on a short read platform (MiSeq, Illumina) and obtained 13,941,057 paired-end (2 × 300-bp) reads to help polish and derive the consensus HytaNPV genome sequence of 139,089 bp (GenBank accession number MH261376) (see Methods). We developed a pipeline (Supplementary Figure S1**)** that identified open reading frames (ORFs) coding for proteins 50 amino acids or longer in the HytaNPV genome and then annotated the ORFs based on the homology to proteins they encode (see Methods). We also used the comparative analysis of genes and gene order in closely related NPV genomes to further refine the annotations.

**Figure 2:**
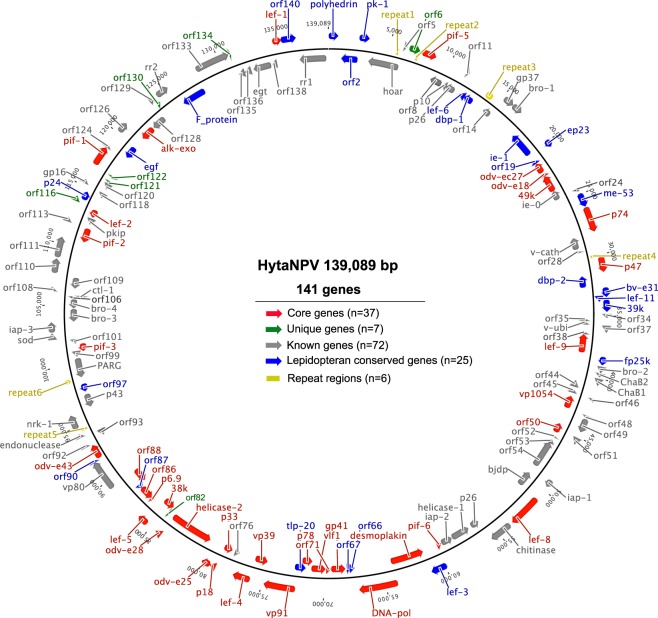
Circular diagram of the HytaNPV genome and annotation. The arrows represent position and direction of ORFs. The first ORF (orf1) is the polyhedrin gene. red – core genes, which are conserved among all baculoviruses; blue – genes conserved among lepidopteran baculoviruses; gray – known genes, which are found in baculovirus; green - unique genes, which are only found in HytaNPV.

The HytaNPV double stranded circular genome is 139,089 bp long and has a GC content of 39.6%. We identified 141 ORFs in the genome that code for proteins of at least 50 amino acids long (Fig. 2). We confirmed the expression of these genes using RNA-seq data derived from HytaNPV infected larvae (Supplementary Table S1 and Supplementary Figure S2). The HytaNPV genome codes for the polyhedrin gene that is observed in all alphabaculoviruses. Keeping with the convention, we designated the polyhedrin gene as *orf1* and used it as the origin to annotate the remaining ORFs. We found that the HytaNPV genome does not code for the envelope fusogenic protein gp64, but contains the coding sequence for the F protein (*orf132*) as observed in group II alphabaculoviruses.

### Phylogeny and genome organization of HytaNPV

**Figure 3:**
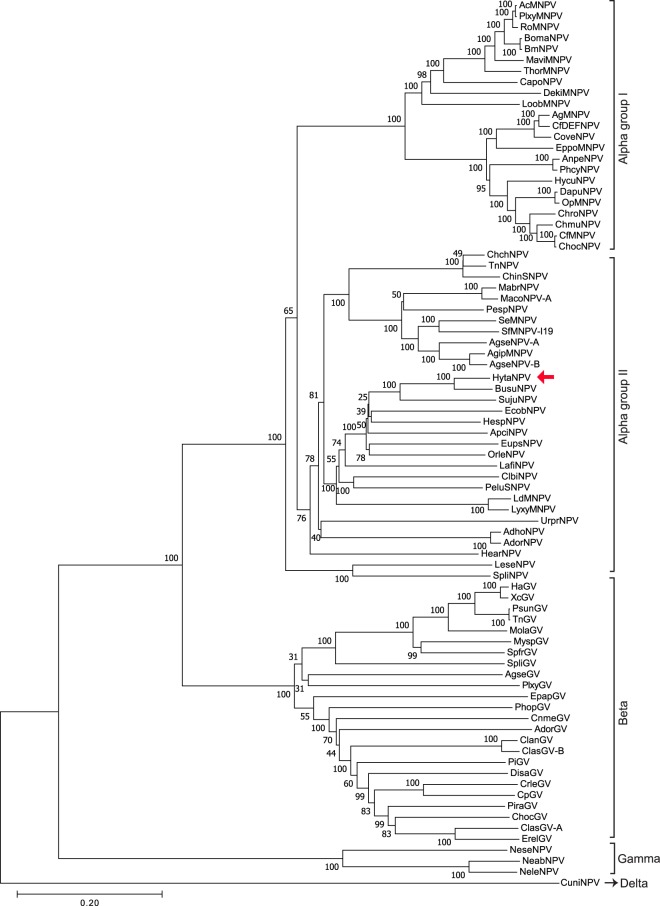
Molecular phylogenetic analysis by Maximum Likelihood method. The tree was constructed using 37 core genes from 81 baculovirus complete genomes (Supplementary Table S4). Bootstrap value resulted from 1000 replications is shown in each node. Red arrow - HytaNPV.

**Figure 4:**
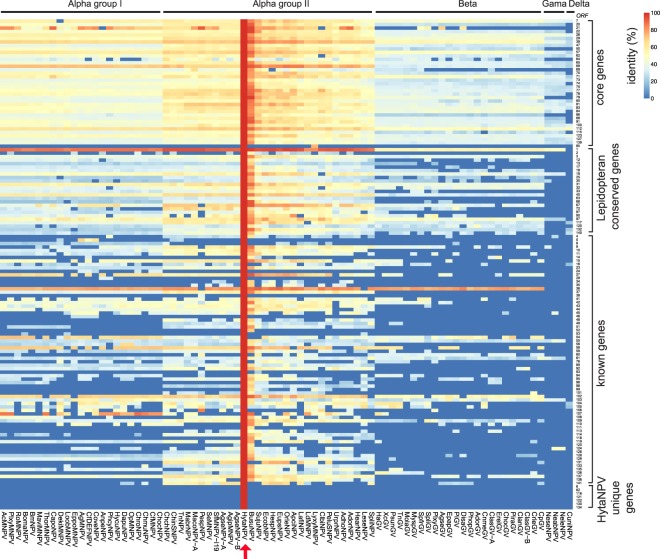
Comparison of HytaNPV against other known baculoviruses. Heatmap showing amino acid identity (%) resulting from blastp (evalue <= 1) of HytaNPV protein sequences against protein sequences of all complete baculovirus genomes. Rows – 141 HytaNPV genes arranged by 4 groups as shown in Fig. 2. Columns – 81 baculoviruses including HytaNPV in the order determined by phylogenetic analysis (Fig. 3). HytaNPV was indicated by red arrow.

We detected all 37 core genes^11^ in the HytaNPV genome that are involved in replication, transcription, oral infectivity, viral assembly, packaging and host protein interactions (Supplementary Table S2). In addition, we analyzed the genome for genes conserved among the four baculovirus subgroups^11^ and found all the 9 genes conserved in alpha, beta and gamma viruses, the F-protein gene conserved in alpha, beta and delta, and all 16 genes conserved in alpha and beta subgroup of baculoviruses (Supplementary Table S3). Phylogenetic analysis based on amino acid sequences of 37 core genes across 81 complete reference genomes (Supplementary Table S2, S4) confirmed that the HytaNPV is a group II alphabaculovirus member (Figs 3–4, Supplementary Figure S3). HytaNPV was closest to *Biston suppressaria* (formerly *Buzura suppressaria*; average amino acid identity between core genes ~76.8%) NPV, though HytaNPV genome is ~19 kb larger. It is interesting to note that *Biston suppressaria*, a moth similar to *Hyposidra talaca* from the Geometridae family, is a known tea looper pest found in China, India and other parts of Asia^12^. The related host niche and the high similarity between the two viruses suggest that they both evolved from a common parent or one is the descendent of the other. In addition, among the next three closely related viruses, two NPVs SujuNPV (average amino acid identity ~57.3%), and EcobNPV (average amino acid identity ~52.8%) also affect geometridae moths, *Sucra jujuba* and *Ectropis obliqua*, respectively. Also, *Ectropis obliqua* is a major tea pest and EcobNPV has been successfully used for its control^13^. This suggests that the host specificity of the baculoviruses probably co-evolved with the insects they infect and often closely related viruses infect larvae from the same family.

**Figure 5:**
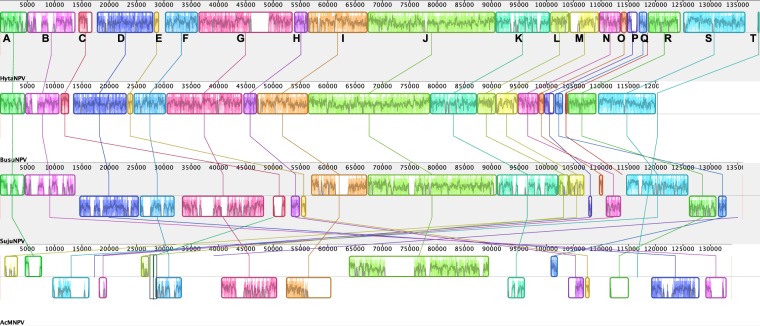
Multiple genome alignment of HytaNPV, BusuNPV, SujuNPV and AcMNPV genomes using progressiveMauve algorithm^14^ with default parameters, where HytaNPV was used as the reference. Conserved genomic regions (locally collinear blocks - LCB) are shown as rectangle blocks with unique colors.

Alignment and analysis of HytaNPV genome to the closely related BusuNPV and SujuNPV genomes, and the prototype AcMNPV genome using Mauve multiple genome alignments software^14^ identified 20 conserved segments shared across these genomes (Fig. 5, Supplementary Table S5). Consistent with the phylogenetic relatedness, HytaNPV genome was collinear with the BusuNPV. Compared to BusuNPV, HytaNPV acquired additional sequences in segment B and G and between segments E-F, L-M and R-S. HytaNPV genome, while very similar in gene content to SujuNPV genome, it shows distinct differences. In particular, segments C to H, N, P, Q, and R are inverted and rearranged in the SujuNPV genome. Interestingly, segment S present in both HytaNPV and SujuNPV encodes ribonucleotide reductase large subunit (rr1) and this is absent in the BusuNPV genome. Comparison of the distantly related AcMNPV and HytaNPV genomes identified segments F, G, I, J and D among the most conserved regions and with the exception of segment J, they are rearranged and inverted. Interestingly, a majority of the conserved blocks carried at least one of the core baculovirus genes with the highly conserved J block encoding 17 of the 37 core genes, indicating an evolutionary constraint that has necessitated the inheritance of these genes as a group during the course of the evolution of the virus (Supplementary Table S2).

### Repeat regions

**Figure 6:**
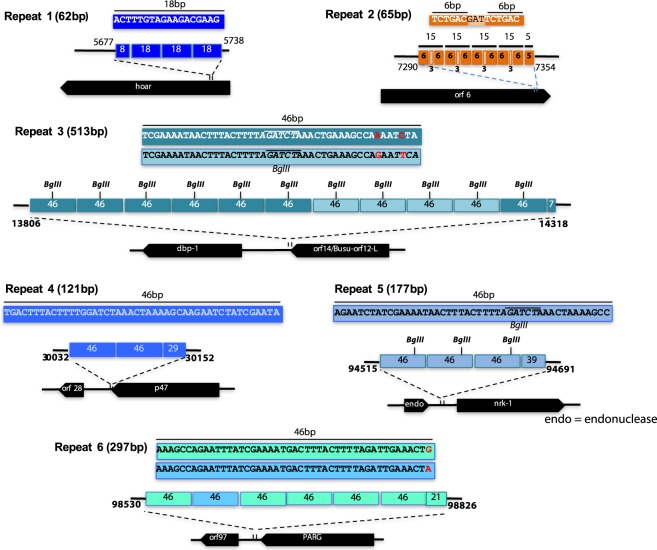
Repeat analysis of HytaNPV. Shown are 6 repeat regions identified in HytaNPV genome.

Repeated A-T rich sequences called homologous repeats (hrs) made of one or more copies of imperfect or perfect palindromic sequence are present in baculovirus genomes^15,16^. They widely vary in the sequence composition, length and number of repeats between genomes^15^. The hrs are thought to function as transcriptional enhancers and replication origins^15^. Similarly, direct repeats (drs) in the baculovirus genomes have been suggested to function as replication origins. In the HytaNPV genome, we found six repeat regions with lengths ranging from 62–513 bp. The repeat sequences were either 46, 18 or 15 bp long and were arranged in tandem (Fig. 6). They were A-T rich with an AT content of 72–74%. Repeat 1, located at the 5p region of hoar (*orf4*), contains three 18 bp repeats and a truncated 8 bp region from the 18 bp unit arranged in tandem. Four 15 bp repeats and a truncated 5 bp region form the 15 bp unit arranged in tandem located at the 3p end of *orf6* constituted repeat 2. Interestingly, the 15 bp unit in repeat 2 consists of two 6 bp repeats with intervening 3 bases separating them. Repeats 3, 4, 5 and 6 were made of 46 bp repeats that shared a common core (Fig. 6), though each 46 bp unit that made each of these repeats was distinct. The repeat 3 is the longest (513 bp) and is made of a total of eleven copies of a 46 bp unit and a truncated 7 bp unit and is located at the 3p end of *orf16*. The 46 bp units in repeat 3 consist of two variants that differ in two positions (Fig. 6). Each of the eleven copies of the 46 bp unit in repeat 3 contains a BglII restriction enzyme site (6 bp) in the middle that is flanked by 20 bp on either side. Repeat 4, located at 3p end of *orf47* is 121 bp long and is made of two 46 bp repeat units and a truncated 29 bp repeat sequence. Between endonuclease (*orf94*) and nrk-1(*orf95*) is the 171 bp repeat 5 that consists of three 46 bp repeat units and a 39 bp truncated repeat sequence. Interestingly the 46 bp repeat units in repeat 5, like repeat 3 units contain a BglII restriction located close to the 3p end of the repeat unit. Repeat 6, 297 bp long, is made of six 46 bp units and a 21 bp truncated repeat sequence. One of the 46 bp unit in repeat 6 differs from the rest by one base at the 3p end (Fig. 6). In the closely related BusuNPV genome a repeat region with two tandem 58 bp repeats is observed^17^. This 58 bp sequence shares a core region observed in the 48 bp units in HytaNPV genome. Previously, 46 bp repeat units in NeleNPV, a gammabaculovirus that infects the hymenopteran *Neodiprion lecontei*, was reported^18^. However, sequence comparison showed that they share very low similarity (Supplementary Figure S4).

### Conserved Noncoding Element (CNE) in HytaNPV

**Figure 7:**
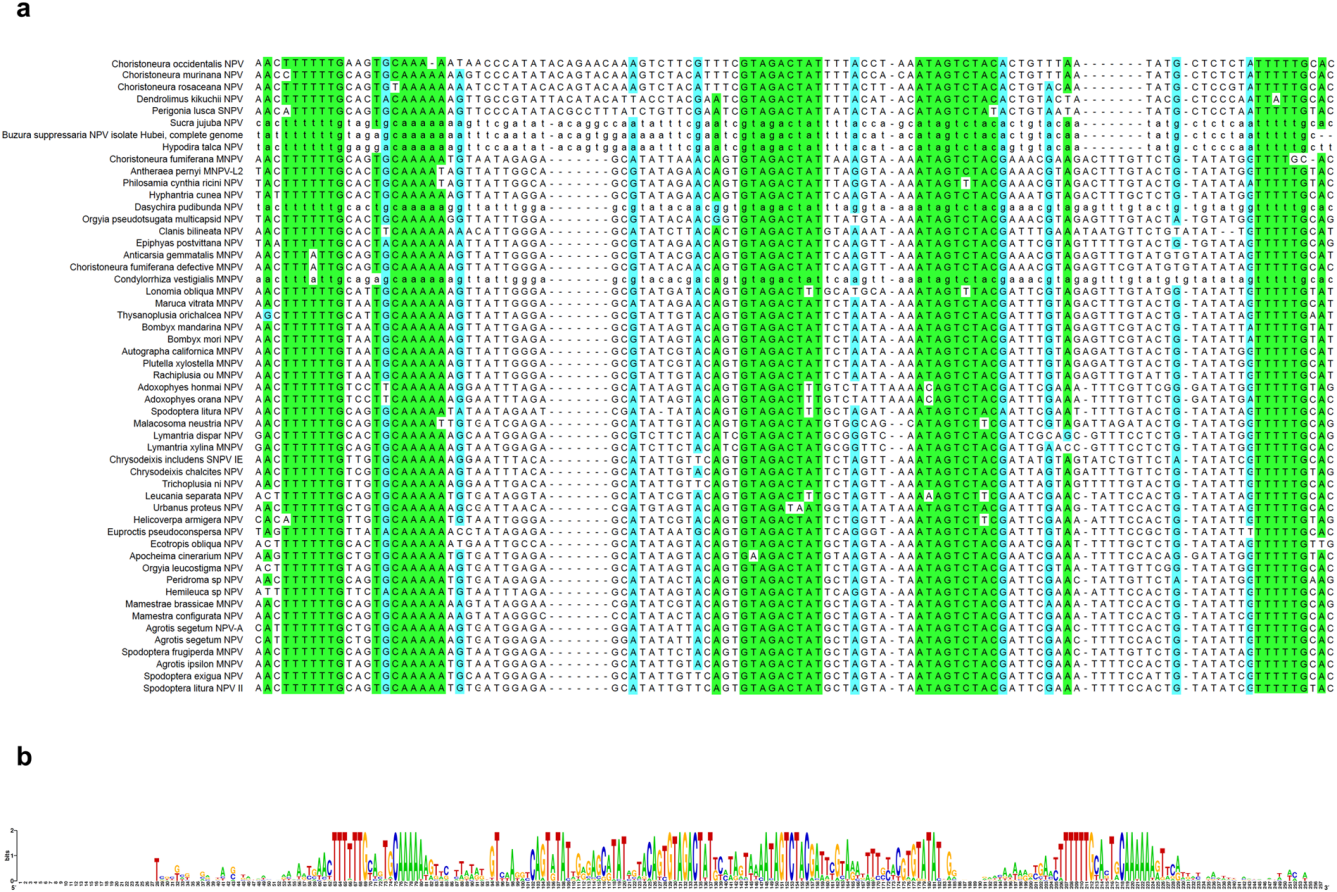
Multiple sequence alignment of Conserved Noncoding Functional Elements (CNEs). (**a**) CNEs identified in 53 alphabaculovirus genomes were aligned using ClustalW. Nucleotides that are identical at a given position across all CNEs are highlighted in light green while those that are identical in at least 70% of all CNEs are highlighted in light blue. (**b**) Consensus CNE sequence from multiple sequence alignment using Weblogo.

We analyzed the HytaNPV genome for the presence of a previously reported, conserved noncoding functional element (CNE) responsible for virus replication in transfected insect cell cultures^19^. This revealed the presence of a CNE in HytaNPV that overlaps with *orf5*. Comparative sequence analysis revealed the presence of CNE in HytaNPV as well as in 52 other alphabaculovirus genomes (Fig. 7). Multiple sequence alignment of CNEs across alphabaculoviruses revealed several highly conserved nucleotide clusters represented by specifically arranged repeat sequences (Fig. 7).

### HytaNPV genes

We analyzed the HytaNPV genome for the presence of genes involved in replication, transcription, viral packaging and other functions based on homology to characterized AcMNPV genes and other sequenced NPV genomes.

A total of 22 genes were identified to be associated with DNA replication based on homology to functionally known genes (Supplementary Table S1, S6). Such genes included DNA polymerase (*DNA-pol* (*orf65*; 1035aa)), alkaline exonuclease (alk-exo (*orf127*; 400aa)), DNA helicases (*helicase-2* (*orf80*; 1238aa) and *helicase-1* (*orf60*; 523aa)), DNA binding proteins (*dbp-1* (*orf13*; 265aa), *dbp-2* (*orf30*; 321aa), *lef-3* (*orf63*; 426aa) and *p6*.*9* (*orf85*; 76aa). It is important to note that the basic protein p6.9 is present in both the closely related SujuNPV and BusuNPV genomes. We also identified genes that code for ribonucleotide reductases, *rr1* (*orf141*; 758aa) and *rr2* (*orf131*; 340aa), and nicotinamide riboside kinase (*nrk-1* (*orf95*; 380aa)) involved in nucleotide biosynthesis. However, in the HytaNPV genome, as in BusuNPV genome, we did not detect a gene for DNA-ligase, though it was found in the closely related SujuNPV genome.

Baculovirus genes can be broadly classified as early, late and very late based on the timing of its expression during the lifecycle of the virus. While the early genes use the host RNA polymerase for its transcription, those expressed late during infection use a virally encoded RNA polymerase. Proteins encoded by *lef-4* (*orf75*; 457aa), *lef-8* (*orf57*; 879aa), *lef-9* (*orf39*; 505aa) and *p47* (*orf29*; 393aa) form the virally encoded RNA polymerase subunits. Additionally, we found six more replication-associated genes, including *vlf1* (*orf68*; 394aa) and *lef-5* (*orf84*; 277aa) that function as initiation factors (Supplementary Table S6). We did not find homologs of *lef-12* and *lef-10*, known to have a role in transcription in the prototype AcMNPV.

In the HytaNPV genome, we identified 39 genes that encode proteins likely involved in viral packaging, viral entry and viral structural integrity. The genes identified included those that code for proteins incorporated into budded virus (BV) and occluded virus, and nucleocapsid. This includes *F protein* (*orf132*; 678aa), *polyhedrin* (*orf1*; 246aa), *p10* (*orf9*; 96aa), and *orf109/Busu-orf99-L/calyx/pep* (*orf109*; 310aa) encoding genes. The F protein (orf132) of HytaNPV has a furin cleavage site and a conserved fusion peptide as observed in group II alphabaculoviruses (Supplementary Figure S5). HytaNPV genome also codes for *orf86/Busu-C42* (*orf86*; 375aa) involved in virus induced actin polymerization^20^.

We identified six genes, *pif-1* (*orf123*; 528aa), *pif-2* (*orf112*; 383aa), *pif-3* (*orf100*; 209aa), *odv-e28/pif-4* (*orf81*; 172aa), *pif-5* (*orf7*; 367aa), and *pif-6* (*orf62*; 121aa), known to be involved in oral infectivity. Further we found genes coding for a viral ubiquitin gene, *v-ubi* (*orf36*; 80aa), a fibroblast growth factor, fgf (*orf128*; 349aa), involved in viral dissemination^21^, the *egt* (*orf140*; 514aa), that codes for ecdysteroid UDP-glucosyltransferase involved in inactivating ecdysone and delaying larval molting^22^, and three inhibitors of apoptosis genes (*iap-1* (*orf55*; 183aa), *iap-2* (*orf61*; 313aa), *iap-3* (*orf103*; 266aa)) that likely function as caspase inhibitors. Interestingly, as with many other baculovirus genomes, the HytaNPV does not code for the p35 caspase inhibitor found in the AcMNPV prototype baculovirus. Also, it does not encode a transcriptional transactivator PE38^23^ (*AcOrf-153*) homolog. However, it encodes (HytaNPV *orf37*) a homolog of ac34 (*AcOrf-34*), that promotes viral replication by blocking chromosomal maintenance 1 (CRM1)-dependent nuclear export^24^.

We found four baculovirus repeat orf (bro) genes whose function is not well characterized. We found 7 ORFs, *orf6* (285aa), *orf82* (57aa), *orf116* (130aa), *orf121* (77aa), *orf122* (68aa), *orf130* (94aa) and *orf134* (51aa), that were unique to HytaNPV. The proteins encoded by these 7 ORFs showed no or very limited homology to known proteins and require further studies to understand their function.

### Genome-wide analysis of HytaNPV promoters

As similar to many DNA viruses, baculovirus genes are transcribed temporally^25^. The early genes mostly use the host transcriptional machinery while the late and very late genes require viral derived protein for their expression^25^.

The core elements of baculovirus early promoters are those recognized by host RNA polymerase II, and sometimes they include the TATA box motif and an initiator sequence (CAGT). Analysis of the prototype baculovirus AcMNPV show that the early gene promoters contain sequence elements recognized by the host RNA polymerase II and typically include a TATA-box-like sequence and CAGT, a transcription initiator (INR) sequence^26,27^. While these elements are typical, they are not always present in all known early gene promoters in AcMNPV^28–30^. The late genes are transcribed by a viral RNA polymerase complex and the transcription is initiated in and around TAAG sequence found within the later promoter^31–34^.

**Figure 8:**
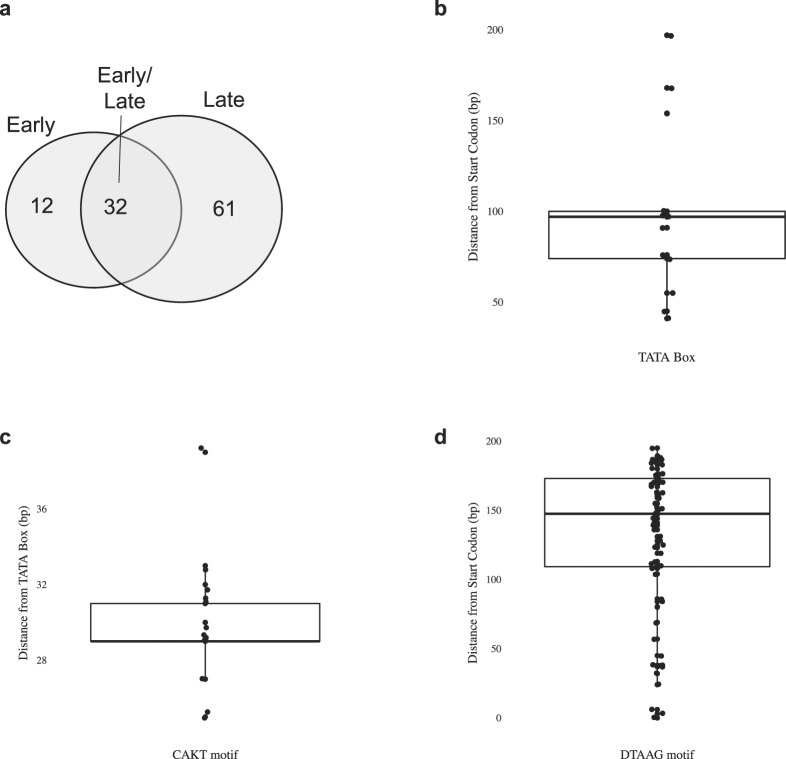
HytaNPV promoter motif analysis. (**a**) Number of predicted early and late (or both) genes in HytaNPV based on the presence of known core promoter motifs in sequences 200 bp immediately upstream of each annotated HytaNPV ORF. (**b**–**d**) Boxplots that show the distribution of distances (in bp) of key core promoter motifs from the start codon (‘ATG’) (**b**,**d**) or TATA box (**b**).

We scanned sequences 200 bp upstream of HytaNPV ORFs for consensus promoter motifs as previously described^32,35^. The significance of these motifs was assessed by comparing their frequencies in sequences downstream of each ORF. Amongst the 141 ORFs in the HytaNPV genome, we identified 12 ORFs that possessed only the early promoter motif (a TATA box linked with a CAG/TT motif ~30 bp downstream), while 61 ORFs had the late promoter motif only (A/T/GTAAG). Thirty two ORFs contained both the early TATA and late TAAG promoter motifs (Fig. 8a; Supplementary Tables S1, S7–9), while the remaining 34 ORFs did not contain any recognizable consensus promoter motifs. We also observed a strong correlation for sequences such as TATAAGG that contain both an early TATA box element and late promoter sequences (ATAAG) in combination. This motif was about ~4 times as frequent in the upstream location compared to sequences downstream of ORFs. TATA sequences combined with the initiator sequences CAGT or CATT separated by ~30-bp were ~3 times as frequent in upstream sequences. Furthermore, 65% of the TATA sequences present in the genome were found to be clustered within 100-bp upstream of the ATG codon of each ORF (Supplementary Table S7), unlike TAAG-containing sequences whose location is more broadly distributed (Fig. 8b–d; Supplementary Table S9). We compared the promoter motifs present in HytaNPV ORFs to those annotated near homologous AcMNPV ORFs and found that ~38% of promoter motifs found in HytaNPV genes were also found near homologous AcMNPV genes (33/86 orthologous ORFs had an identical promoter motif class (E, L or E/L)). Additionally, we compared upstream sequences of HytaNPV ORFs that had homologs in two closely related genomes (BusuNPV, SujuNPV) to identify conserved promoter motifs, including a well-known late baculovirus gene, *polyhedrin*. This analysis revealed a strong conservation promoter motifs upstream of homologous ORFs (Supplementary Figure S6 and Supplementary Table S1).

Analysis of RNA-seq data obtained from the gut tissue of *H*. *talaca* infected larvae (see Method) showed that 134/141 (95%) HytaNPV ORFs are expressed (RPKM > 2) at either 24 h or 72 h (Supplementary Table S1, Supplementary Figure S2). We further examined the concordance between the promoter motif prediction and the RNA-seq data. Out of 12 ORFs with an early-promoter motif, 4 ORFs expressed higher at early time point (24 h) (Supplementary Table S1). This includes *p43* (orf96) and *egt* (orf137), which are also known to express early during AcMNPV infection^36,35^. Among 62 ORFs containing a late-promoter motif, 31 were found elevated late (72 h) during the infection cycle (Supplementary Table S1). As expected, *Polyhedrin* (orf1), a gene known to be expressed late during infection showed a maximum expression at 72 h (Supplementary Table S1). This is consistent with the expression pattern of *Polyhedrin* (AcOrf-8)^6^ observed in AcMNPV^35^. Among 32 ORFs with both the early- and late-promoter motifs, 29 ORFs (~91%) were found to be highly expressed at both 24 h and 72 h (RPKM > 200). These data confirmed the temporal expression of the HytaNPV genes during the infection cycle.

## Discussion

We have obtained the complete sequence of the HytaNPV genome using single molecule sequencing. We found that it belongs to the group II alphabaculoviruses and is closely related to BusuNPV and SujuNPV. The elucidation of the complete sequence of this virus will enable the development of specific PCR based tests that will support the development of a HytaNPV based biopesticide. Such a biopesticide will help reduce the use of chemical pesticides in tea plantations and enhance the quality of tea produced in *H*. *talaca* infested areas.

## Materials and Methods

### Samples

We obtained NPV infected *H*. *talaca* larvae from tea fields of Dooars region of West Bengal, India. Infected larvae were macerated using a sterile mortar and pestle in distilled water. Large particulates and insect debris were removed by centrifugation at 150 g at 4 °C for 5 minutes. Polyhedral inclusion bodies (PIBs) in the supernatant were then pelleted by centrifugation at 20,000 g for 10 min at 4 °C. The PIBs were washed in distilled water three times and pelleted to remove any additional particulate materials. PIBs were characterized using a compound microscope and used for further studies.

### Scanning Electron Microscopy (SEM)

A 2.5ul drop of the aqueous PIB suspension were placed on Formvar and carbon-coated TEM grid and adsorbed to the grid for 30 min at RT. The grids were then quickly rinsed with water, negatively stained with 1% uranyl acetate for 30 sec and finally air-dried. Negatively stained PIBs were examined in a JEOL JEM-1400 transmission electron microscope (TEM) at 80 kV. Digital images were captured with a GATAN Ultrascan 1000 CCD camera at magnifications from 3000× to 15000×.

### Sequencing and assembly

Sequencing was done using RSII Pacific Biosciences single molecule platform. Read processing and de novo assembly were performed using HGAP Assembly workflow (version 2.0)^37^ as implemented in Pacbio SMRT Portal. Illumina MiSeq short read data were used to polish the PacBio assembly. Briefly, we mapped MiSeq reads onto PacBio assembly sequence using BWA-MEM (version 0.7.10) and called variants using GATK Haplotype Caller (version v3.5)^38^. High-confidence variants, which meet criteria recommended by GATK^39^, were used for manual correction to derive the consensus HytaNPV assembly.

### Gene annotation

We developed a pipeline for calling and annotating ORFs as outlined in Supplementary Figure S1 (code available upon request). Briefly, hypothetical open reading frames (ORFs) were predicted using EMBOSS sixpack program^40^, with at least 50 amino acids. For any pair of ORFs that are overlapped (>40%) in any orientation, the longer ORF was kept and the shorter ORF was discarded if it is not known in baculoviruses. ORFs are reviewed and annotated using NCBI Protein-Protein BLAST algorithm (version 2.2.30+).

### RNA-seq

*H*. *talaca* larvae were reared in laboratory from the eggs obtained from a single moth. The larvae were reared on tea leaf. Third instar larvae were placed on tea leaf and sprayed with HytaNPV particles (1 × 10^10^ PIBs/ml). Three larvae each were dissected at 0 h, 24 h and 72 h to obtain the gut tissue. Total RNA was isolated from pooled gut tissue corresponding to each time point using Trizol (Thermo Fisher Scientific). Libraries were prepared using TruSeq® stranded total RNA library prep kit (Illumina) using 1 μg of total RNA as starting material. The final libraries obtained were sequenced on Illumina HiSeq 2500 platform to obtain ~20 million 2 × 100 bp paired end reads for each library.

The reads were mapped to HytaNPV genome sequence using Bowtie (version 0.12.9) allowing for one mismatch within the 60 bp high-quality end of the reads. We observed 3 reads (at 0 h), 17,342 reads (at 24 h), and 57,040 reads (at 72 h) mapped uniquely to HytaNPV genome. Uniquely mapped reads at 24 h and 72 h time points were used to quantify expression (reads per kilobase of exon model per million mapped reads (RPKM)) of HytaNPV genes.

### Conserved noncoding element (CNE) detection

Blastn^41^ was performed to identify the previously described CNE^19^ with parameters – word size = 7, match/mismatch = 1,−1, gap existence/extend = 5,2. The top hit from Blastn for all sequenced alphabaculovirus was then collected and supplied as input to the ClustalW^42^ program to identify highly conserved clusters within homologous CNEs from each baculovirus species. Finally, Weblogo^43^ was used to generate a consensus CNE sequence across all Alphabaculoviruses.

### Promoter motif analysis

Genome sequences 200 bp upstream of each HytaNPV ORF were extracted and searched for known consensus core promoter motifs, namely, TATAA, A/T/GTAAG, and CAG/T using multiple expectation maximization for motif elicitation (MEME)^44^. Parameter 0 or 1 motif per sequence and an E value of 0.001 were used.

### Phylogenetic analysis

Phylogenetic analysis was based on amino acid sequences of 37 core genes extracted from 81 complete baculovirus genomes (Supplementary Table S4). All the sequences were aligned using the multiple sequence alignment algorithm clustalW (version 2.1)^42^ with default parameters. A phylogenetic tree was constructed using Maximum Likelihood method with Jones-Taylor-Thornton (JTT) model^45^ as implemented in the MEGA7 software^46^. Phylogeny test was carried out using Bootstrap method with 1000 replicates.

### Availability of data and materials

The HytaNPV genome sequence is available from GenBank under accession number MH261376.

## Electronic supplementary material

Supplementary figures S1-S6

Supplementary tables S1-S9
