# Author Correction: Comprehensive analysis of single molecule sequencing-derived complete genome and whole transcriptome of *Hyposidra talaca* nuclear polyhedrosis virus

**DOI:** 10.1038/s41598-018-31454-x

**Published:** 2018-08-31

**Authors:** Thong T. Nguyen, Kushal Suryamohan, Boney Kuriakose, Vasantharajan Janakiraman, Mike Reichelt, Subhra Chaudhuri, Joseph Guillory, Neethu Divakaran, P. E. Rabins, Ridhi Goel, Bhabesh Deka, Suman Sarkar, Preety Ekka, Yu-Chih Tsai, Derek Vargas, Sam Santhosh, Sangeetha Mohan, Chen-Shan Chin, Jonas Korlach, George Thomas, Azariah Babu, Somasekar Seshagiri

**Affiliations:** 1 Department of Molecular Biology, Genentech Inc., 1 DNA WAY, South San Francisco, CA 94080 USA; 2 AgriGenome Labs Private Limited, 501, SCK01 Building, SmartCity Kochi, Infopark Road, Kakkanad, Kochi, Kerala 682 042 India; 3 SciGenom Research Foundation, 3rd Floor, Narayana Health City, #258/A, Bommasandra, Hosur Road, Bangalore, Karnataka 560 099 India; 4 Department of Pathology, Genentech Inc, 1 DNA WAY, South San Francisco, CA 94080 USA; 5 Tea Research Association, North Bengal Regional R & D Centre, Nagrakata, Jalpaiguri, West Bengal 735 225 India; 6 Pacific Biosciences, 1305O’Brien Dr, Menlo Park, CA 94025 USA; 7 SciGenom Labs Pvt Ltd, Plot no: 43A,SDF, 3rd floor, A Block, CSEZ, Kakkanad, Kochi, Kerala 682 037 India

Correction to: *Scientific Reports* 10.1038/s41598-018-27084-y, published online 12 June 2018

In the original version of this Article, there were errors in Affiliations 2, 3 and 7 which were incorrectly listed as ‘AgriGenome Labs Private Limited, 501, SCK01 Building, Smart City Kochi, Infopark Road, Kakkanad, Kochi, 682 030, India’, ‘SciGenom Research Foundation, 3rd Floor, Narayana Health City, #258/A, Bommasandra, Hosur Road, Bangalore, 560 099, India’ and ‘SciGenom Labs Pvt Ltd, Plot no: 43A,SDF, 3rd floor, A Block, CSEZ, Kakkanad, Cochin, Kerala, 682 037, India’ respectively.

The correct affiliations are listed below:

Affiliation 2

AgriGenome Labs Private Limited, 501, SCK01 Building, SmartCity Kochi, Infopark Road, Kakkanad, Kochi, Kerala, 682 042, India

Affiliation 3

SciGenom Research Foundation, 3rd Floor, Narayana Health City, #258/A, Bommasandra, Hosur Road, Bangalore, Karnataka, 560 099, India

Affiliation 7

SciGenom Labs Pvt Ltd, Plot no: 43A,SDF, 3rd floor, A Block, CSEZ, Kakkanad, Kochi, Kerala, 682 037, India

These errors have now been corrected in the PDF and HTML versions of the Article, and in the Supplementary Material files.

