# Supplementary material for: Comprehensive analysis of single molecule sequencing-derived complete genome and whole transcriptome of Hyposidra talaca nuclear polyhedrosis virus
Source: Sci Rep. 2018 Jun 12;8:8924. doi: 10.1038/s41598-018-27084-y (PMC5997678; doi:10.1038/s41598-018-27084-y)

Supplementary figures:

Comprehensive analysis of single molecule sequencing-derived complete genome and whole transcriptome of *Hyposidra talaca* nuclear polyhedrosis virus

Thong T. Nguyen[1], Kushal Suryamohan[1], Boney Kuriakose[2,3], Vasantharajan Jankiraman[1], Mike Reichelt[4], Subhra Chaudhuri[1], Joseph Guillory[1], Neethu Divakaran[2], Rabins PE[2], Ridhi Goel[2], Bhabesh Deka[5], Suman Sarkar[5], Preety Ekka[5], Yu-Chih Tsai[6], Derek Vargas[1], Sam Santhosh[7], Sangeetha Mohan[7], Chen-Shan Chin[6], Jonas Korlach[6], George Thomas[2,3], Azariah Babu[5] and Somasekar Seshagiri[1*]

[1]Department of Molecular Biology, Genentech Inc., 1 DNA WAY, South San Francisco, CA, 94080, USA; [2]AgriGenome Labs Private Limited, 501, SCK01 Building, SmartCity Kochi, Infopark Road, Kakkanad, Kochi, Kerala, 682 042, India; [3]SciGenom Research Foundation, 3rd Floor, Narayana Health City, #258/A, Bommasandra, Hosur Road, Bangalore, Karnataka, 560 099, India; [4]Department of Pathology, Genentech Inc., 1 DNA WAY, South San Francisco, CA, 94080, USA; [5]Tea Research Association, North Bengal Regional R & D Centre, Nagrakata, Jalpaiguri, West Bengal, 735 225, India; [6]Pacific Biosciences, 1305 O'Brien Dr, Menlo Park, CA, 94025, USA; [7]SciGenom Labs Pvt Ltd, Plot no: 43A,SDF, 3rd floor, A Block, CSEZ, Kakkanad, Kochi, Kerala, 682 037, India.

*Correspondence. S.S. sekar@gene.com; phone: 650-225-1000; fax: 650-225-1762

Supplementary Figure legends

Supplementary Figure S1: Flowchart showing pipeline used for ORFs detection and annotation of the HytaNPV genome.

Supplementary Figure S2: Expression of HytaNPV ORFs at 24h and 72h using RNA-seq data.

Supplementary Figure S3: Comparison of HytaNPV against other baculoviruses used for biopesticides. Heatmap showing amino acid identity (%) resulting from blastp (evalue <= 1) of HytaNPV protein sequences against protein sequences of all complete baculovirus genomes.

Supplementary Figure S4: Alignment of repeat sequences from HytaNPV and that observed in BusuNPV.

Supplementary Figure S5: Alignment of F and F-like proteins across baculoviruses. The multiple-sequence alignment was carried out using Clustal Omega[1] and visualized using Jalview software[2]. Cartoon of conserved regions (SP – signal peptide; FP – fusion peptide; S-S disulfide bridge; TM – transmembrane domain) of SeMNPV F protein shown at the bottom was adapted from Westenberg et al.[3]. Order of protein sequences appear on the left was determined by Clustal Omega's algorithm. A1 – Alphabaculovirus group I, B – Betabaculovirus; A2 – Alphabaculovirus group II. Amino acid conservation was highlighted in blue.

Supplementary Figure S6: Alignment of DNA sequences upstream of *polyhedrin* gene of AcMNPV, BusuNPV, SujuNPV and HytaNPV showing a conserved core promoter region.

Reference:

1 Sievers, F. *et al.* Fast, scalable generation of high-quality protein multiple sequence alignments using Clustal Omega. *Mol Syst Biol* **7**, 539, doi:10.1038/msb.2011.75 (2011).

2 Clamp, M., Cuff, J., Searle, S. M. & Barton, G. J. The Jalview Java alignment editor. *Bioinformatics* **20**, 426-427, doi:10.1093/bioinformatics/btg430 (2004).

3 Westenberg, M. *et al.* Functional analysis of the putative fusion domain of the baculovirus envelope fusion protein F. *J Virol* **78**, 6946-6954, doi:10.1128/JVI.78.13.6946-6954.2004 (2004).

Supplementary Figure S1

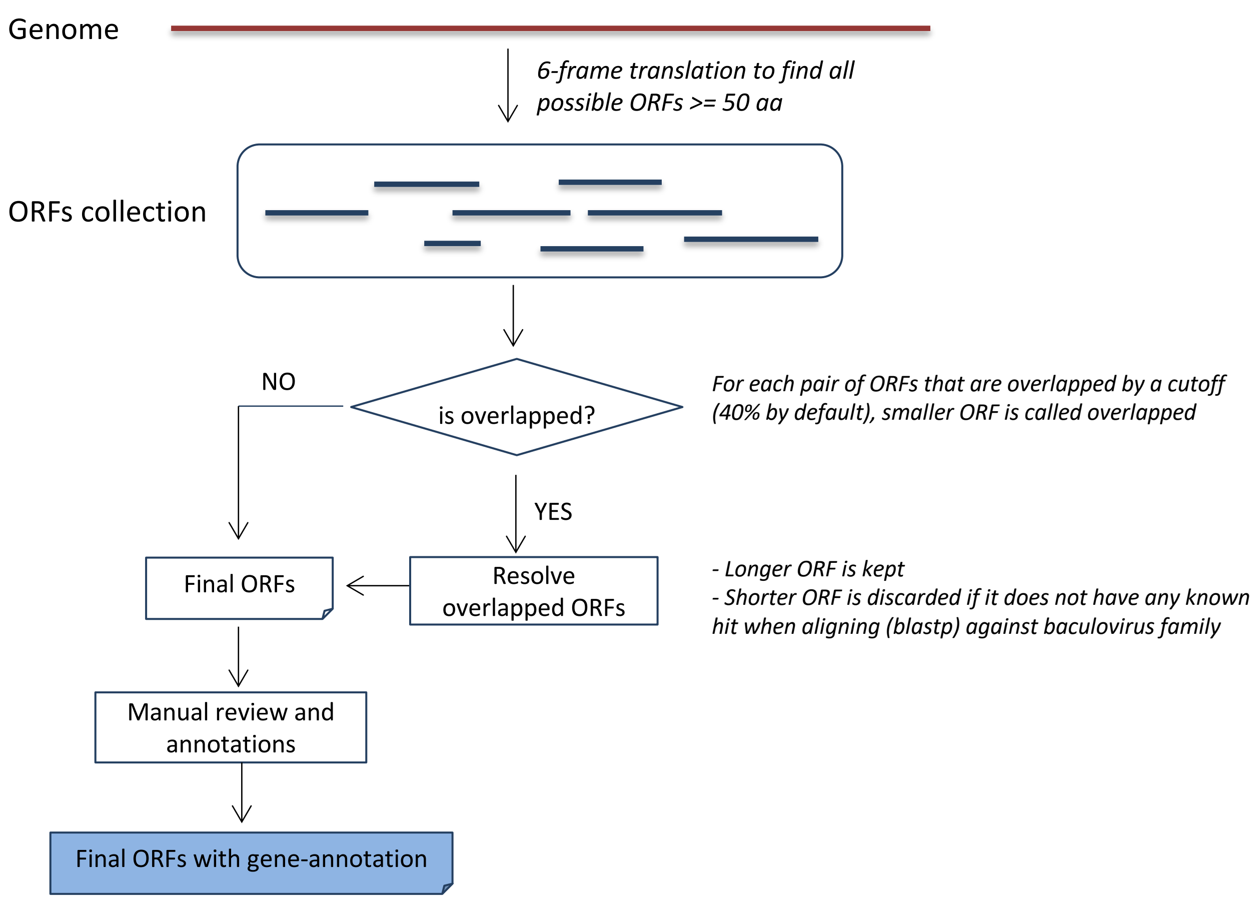

Supplementary Figure S2

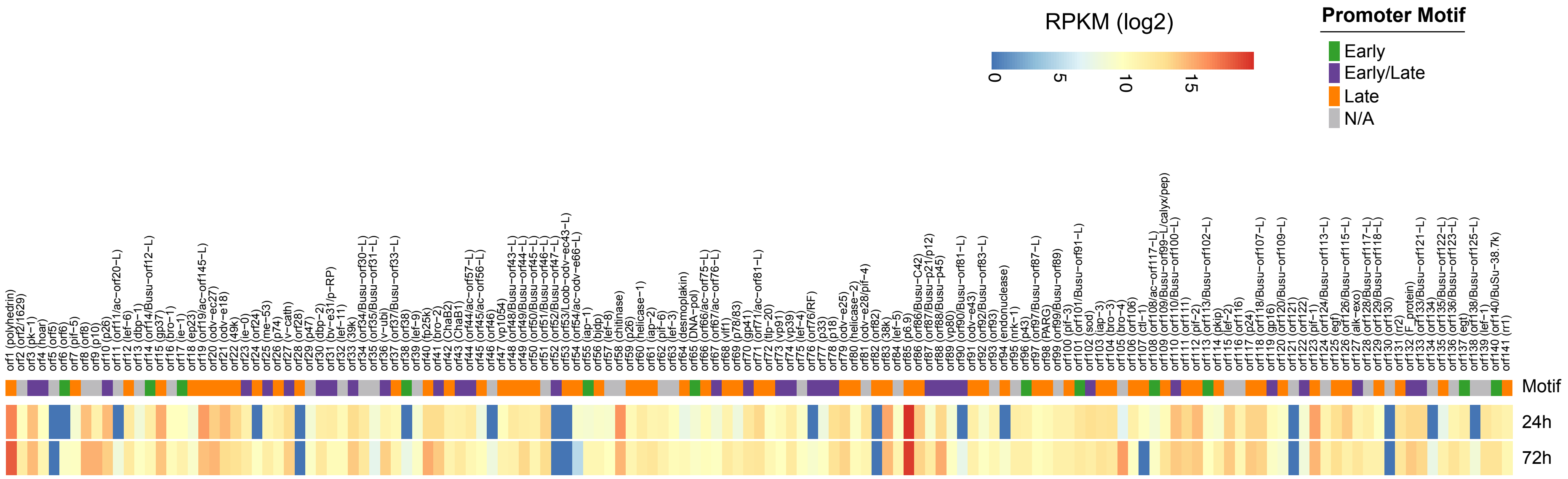

Supplementary Figure S3

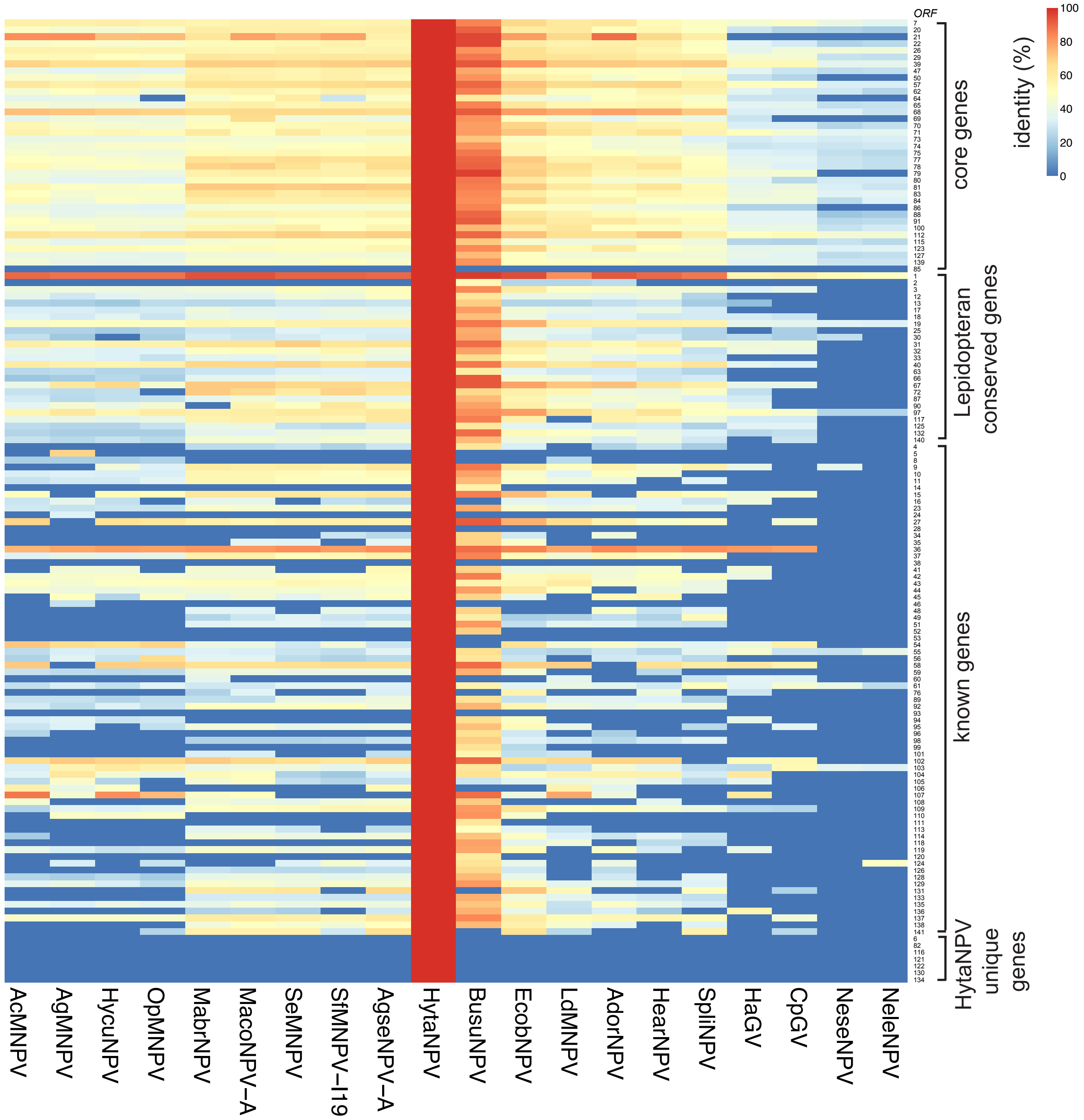

Supplementary Figure S4

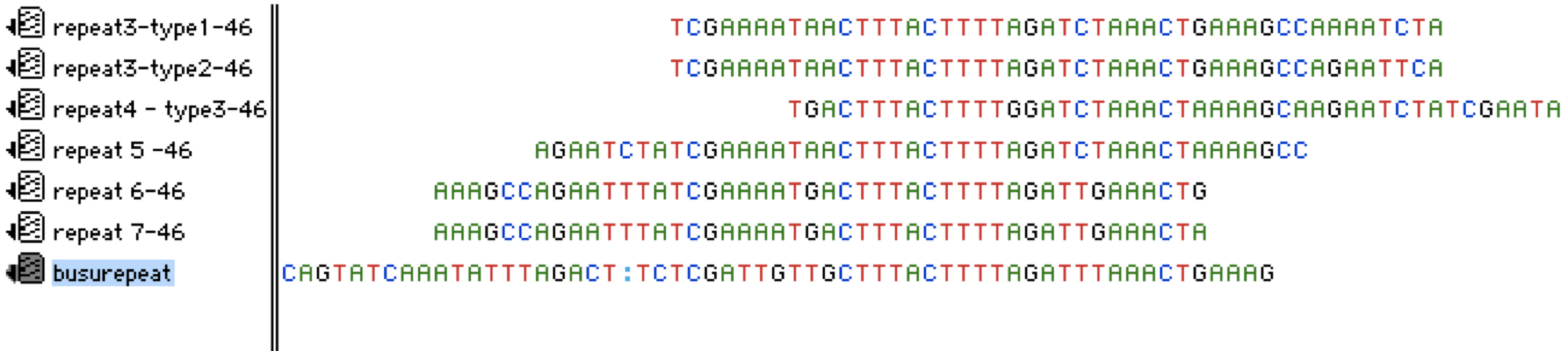

Supplementary Figure S5

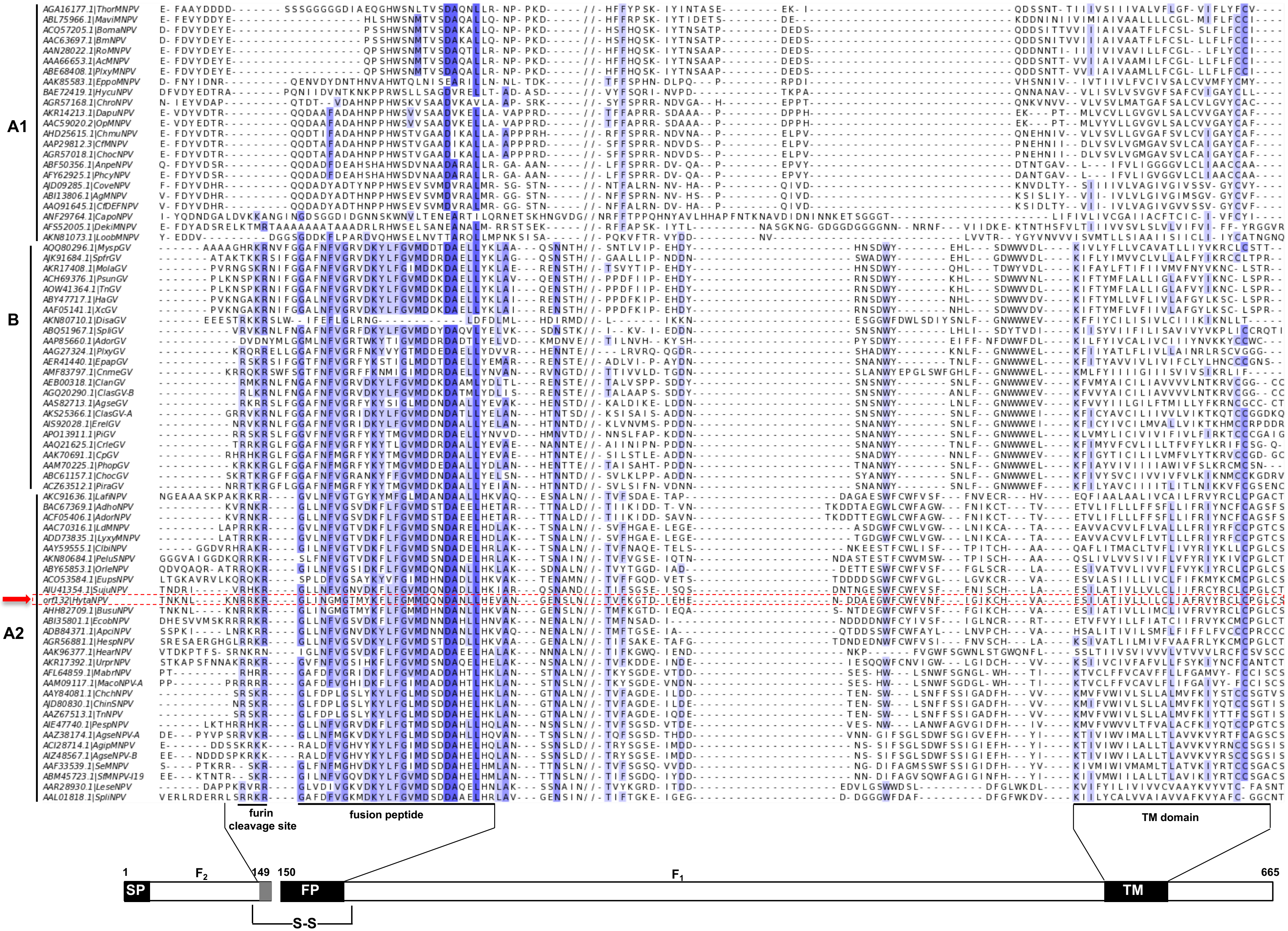

Supplementary Figure S6

```
Reference sequence (1): AcMNPV
Identities normalised by aligned length.
Colored by: identity + property

                              1 [         .         .         .         .         :         .         .         .
 1 AcMNPV         100.0%        actattgtctgcgagcagttgtttgt--tgttaaa-aataacagccattgtaatgagacgc--acaaactaatatcac--
 2 BusuNPV         48.6%        -cttttta-atgctgcctgttgcacat--cgatctgttgtacat---gttgaaacg-gatg-agat--ctcaatatttga
 3 HytaNPV         50.0%        -attgta-acgcgtactgtcgt-cgt--cacactgcagttaaa---attaacattacatg-atattatgcattatcataa
 4 SujuNPV         48.3%        ----tta-atgtaaat--ttgtacgtgagaaaatataataaaaaccttgatactacttgtattgttttgatttt-tta
   consensus/100%               ....sTu.ssGsssss..TsGs.suT..sussssu.suTssss...sTTussAss.sssG...ss..sssusTsTs.s..
   consensus/90%                ....sTu.ssGsssss..TsGs.suT..sussssu.suTssss...sTTussAss.sssG...ss..sssusTsTs.s..
   consensus/80%                ....sTu.ssGsssss..TsGs.suT..sussssu.suTssss...sTTussAss.sssG...ss..sssusTsTs.s..
   consensus/70%                 sTsTTA ATGCuuuCsGTTGTsCGT  susssTusAuTAAAA...uTTGAsAssAsATG.AsAssssssAATATssTsA

                             81         .         1         .         .         .         .         :         .
 1 AcMNPV         100.0%        aaactggaaatgtc-tatcaata-tatagttgct-gatatcatggag---ataat-taaaatgataaccatctcgcaa--
 2 BusuNPV         48.6%        acacctg--acggctttacagca-tgtaattgca-tatacgatgttttataaattatcaaaatttgttcatt-tacaatc
 3 HytaNPV         50.0%        atactgg--acggcttatcagtg---cgctcgctctatacgatgttttgtaaatt-ttaaatttgttcatt-tacaaac
 4 SujuNPV         48.3%        aaactat--atgtat-cccatgacaataatcgat-tatatgattaattgtaaaat-ttaaatt-cttcagt-cacaacg
   consensus/100%               AsACsss..AsGss..ssCAssu...susTsGss.sATAssATssss...AsAsT.TsAAAsss.sssCAss.suCAA..
   consensus/90%                AsACsss..AsGss..ssCAssu...susTsGss.sATAssATssss...AsAsT.TsAAAsss.sssCAss.suCAA..
   consensus/80%                AsACsss..AsGss..ssCAssu...susTsGss.sATAssATssss...AsAsT.TsAAAsss.sssCAss.suCAA..
   consensus/70%                AsACTuG  AsGsCTTssCAusA suTAuTsGCT TATAsGATGssTTuTAAAsT TsAAAsTTTuTTCATT sACAAss

                            161         .         .         .         2         .      ] 217
 1 AcMNPV         100.0%        ataaataagtattttactgttttcgtaacagttttgtaataa-aaaaacctataaat
 2 BusuNPV         48.6%        ttcaataagtattttttcctattgtaaaaca-ttgtgaaaaatcaaatacaacata
 3 HytaNPV         50.0%        ttcaataagtattttttcctattgtaaaaca-ttgtgaaaaatcaaatataacata
 4 SujuNPV         48.3%        ttcaataagtattttgttctattgtaaaaca-ttgtgaaaaatcaaatac-acata
   consensus/100%               sTsAATAAGTATTTTssTssTsTsGTAAsAss.TTGTuAsAA.ssAAAsss.ssAss
   consensus/90%                sTsAATAAGTATTTTssTssTsTsGTAAsAss.TTGTuAsAA.ssAAAsss.ssAss
   consensus/80%                sTsAATAAGTATTTTssTssTsTsGTAAsAss.TTGTuAsAA.ssAAAsss.ssAss
   consensus/70%                TTCAATAAGTATTTTTsTsCTATTGTAAAACA TTGTGAAAAATCAAATAsAACATA
```

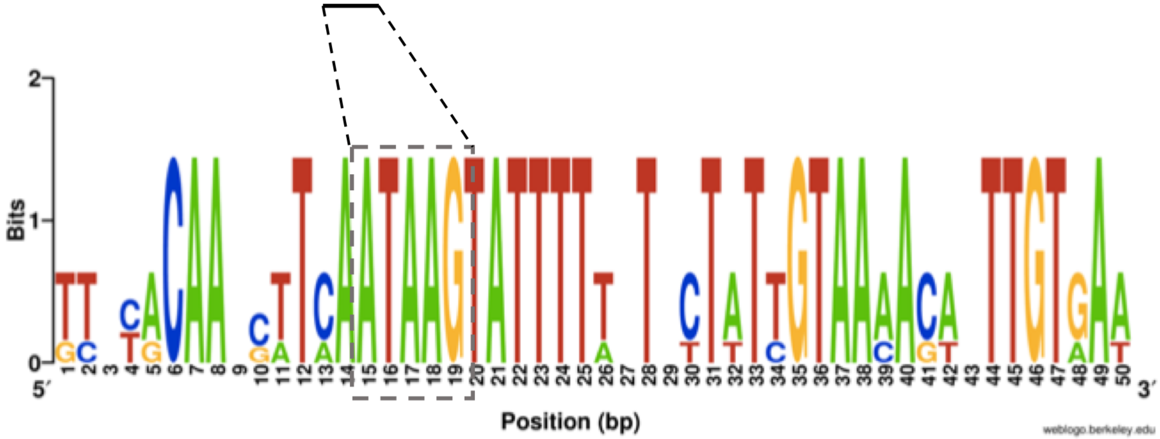

Supplement: Supplementary file 1 — Supplementary figures S1-S6 [file 41598_2018_27084_MOESM1_ESM.pdf]
